# Current Approaches and Innovations in Managing Preeclampsia: Highlighting Maternal Health Disparities

**DOI:** 10.3390/jcm14041190

**Published:** 2025-02-11

**Authors:** Alexis G. Dickerson, Christiana A. Joseph, Khosrow Kashfi

**Affiliations:** 1 Department of Molecular, Cellular, and Biomedical Sciences, Sophie Davis School of Biomedical Education, City University of New York School of Medicine, New York, NY 10031, USA; adicker000@citymail.cuny.edu (A.G.D.); cnunez2301@baypath.edu (C.A.J.); 2 Department of Chemistry and Physics, State University of New York at Old Westbury, Old Westbury, NY 11568, USA; 3 Graduate Program in Biology, City University of New York Graduate Center, New York, NY 10091, USA

**Keywords:** preeclampsia, pregnancy, maternal mortality, nitric oxide, hydrogen sulfide, carbon monoxide, aspirin, statins, racial disparities

## Abstract

Preeclampsia (PE) is a major cause of maternal mortality and morbidity, affecting 3–6% of pregnancies worldwide and ranking among the top six causes of maternal deaths in the U.S. PE typically develops after 20 weeks of gestation and is characterized by new-onset hypertension and/or end-organ dysfunction, with or without proteinuria. Current management strategies for PE emphasize early diagnosis, blood pressure control, and timely delivery. For prevention, low-dose aspirin (81 mg/day) is recommended for high-risk women between 12 and 28 weeks of gestation. Magnesium sulfate is also advised to prevent seizures in preeclamptic women at risk of eclampsia. Emerging management approaches include antiangiogenic therapies, hypoxia-inducible factor suppression, statins, and supplementation with CoQ10, nitric oxide, and hydrogen sulfide donors. Black women are at particularly high risk for PE, potentially due to higher rates of hypertension and cholesterol, compounded by healthcare disparities and possible genetic factors, such as the *APOL1* gene. This review explores current and emerging strategies for managing PE and addresses the underlying causes of health disparities, offering potential solutions to improve outcomes.

## 1. Introduction

Each year, 3–6% of pregnancies worldwide are affected by preeclampsia (PE) [1]. In the United States, PE is a leading cause of maternal mortality and morbidity [2]. This pregnancy complication is characterized by hypertension, typically accompanied by proteinuria, arising after the 20th week of gestation [3,4]. However, PE may also be diagnosed in the absence of proteinuria if the patient shows symptoms of end-organ dysfunction [5,6]. PE can lead to severe consequences, including placental ischemia, increased arterial resistance, maternal endothelial dysfunction, renal failure, eclamptic seizures, and maternal or fetal death [3,7]. The precise cause of PE remains unknown, but current consensus suggests that its origin lies in abnormal placental development. In healthy pregnancies, cytotrophoblast invasion of the uterine wall transforms uterine spiral arteries into a low-resistance vascular system, facilitating the transfer of oxygen and nutrients from mother to fetus [8]. In PE, however, deficient cytotrophoblast invasion and the insufficient transformation of spiral arteries lead to placental ischemia and the release of antiangiogenic factors [9]. These antiangiogenic factors, sFlt-1 (soluble fms-like kinase 1) and sEng (soluble endoglin), inhibit blood vessel formation in the placenta, resulting in elevated blood pressure [10]. As sFlt-1 and sEng increase, they reduce levels of proangiogenic factors such as vascular endothelial growth factor (VEGF) and placental growth factor (PIGF) [11]. In addition, elevated oxidative stress and reactive oxygen species (ROS) in both the placenta and maternal circulation are commonly observed in PE, contributing to endothelial dysfunction [12,13]. Other factors associated with PE include increased hypoxia-inducible factor 1α (HIF-1α), reduced levels of physiological gasotransmitters (nitric oxide (NO), carbon monoxide (CO), and hydrogen sulfide (H_2_S)), and elevated levels of coenzyme Q10 (CoQ10) [1,14,15].

Various maternal risk factors also correlate with PE, such as age, parity, pregnancy interval, family history, obesity, comorbidities, ethnicity, and race [16]. Despite substantial progress in understanding the pathophysiology of PE, significant challenges remain in terms of early detection, prevention, and optimal treatment. Current management strategies primarily focus on symptom management rather than addressing the underlying mechanisms, and there is a need for improved therapeutic interventions. Furthermore, while racial and ethnic disparities in PE outcomes are well documented, their mechanisms are not fully understood, and strategies to address these disparities remain underexplored. This review aims to provide a comprehensive summary of the current understanding of PE, with an emphasis on the mechanisms underlying the condition, current management strategies, and emerging therapeutics. Additionally, it explores the impact of health disparities on PE outcomes, offering insights into potential avenues for reducing these disparities and improving maternal and fetal health outcomes across diverse populations.

## 2. Diagnostic Measures

### 2.1. Historical Perspectives

The understanding and diagnostic criteria for PE have evolved significantly over the decades. In the 1950s, William Dieckmann, a professor at the University of Chicago, provided one of the first detailed descriptions of the pathogenesis and diagnostic criteria for what was then referred to as “toxemia” [17,18,19]. He defined PE as the presence of edema, proteinuria exceeding 0.3 g per 24 h for at least three days, and hypertension with blood pressure readings above 140/90 mmHg [17]. In 1972, the American College of Obstetrics and Gynecology (ACOG) formalized this definition, specifying PE as hypertension (>140/90 mmHg) and proteinuria (0.3 g per 24 h for >3 days) or edema occurring after 20 weeks of gestation [17]. In 1988, Dennis Davey and Ian MacGillivray introduced a nuanced classification for pregnancy-induced hypertension. They categorized cases into three groups: gestational hypertension without proteinuria, gestational proteinuria without hypertension, and gestational proteinuria with hypertension [18]. Through their research, they observed an increased rate of proteinuria alongside a diastolic blood pressure rise of at least 30 mmHg. However, they concluded that proteinuria alone should not be a defining diagnostic criterion for PE [17]. By the early 2000s, significant changes were made to the diagnostic framework. The criterion of edema was removed, recognizing that it was a common feature even among healthy pregnancies and lacked specificity for PE [17]. In 2000, the Australian Society for the Study of Hypertensive Disorders in Pregnancy (ASSHP) expanded diagnostic measures to include organ dysfunction beyond proteinuria, such as hematological, hepatic, and renal abnormalities [17,19]. However, the 2002 ACOG guidelines remained more conservative, still emphasizing elevated blood pressure and proteinuria as the primary diagnostic markers for PE. Organ dysfunction was classified under “severe preeclampsia” at this time [17,20]. A pivotal shift occurred in 2013 when ACOG broadened the diagnostic criteria for PE, recognizing that proteinuria and hypertension alone were insufficient. The revised guidelines acknowledged that organ dysfunction, such as thrombocytopenia, impaired liver function, renal insufficiency, pulmonary edema, or visual disturbances, could be diagnostic indicators, even in the absence of proteinuria [21]. Proteinuria diagnostic thresholds were defined as the excretion of 300 mg or more of protein in a 24 h collection, a protein/creatinine ratio of ≥0.3 mg/dL, or a dipstick reading of +1. Importantly, these updates emphasized that not all patients with PE exhibit proteinuria, underscoring the importance of recognizing systemic organ dysfunction in the diagnostic process.

### 2.2. Diagnostic Measures and Current Management

The current diagnostic criteria for PE, as outlined by the American College of Obstetricians and Gynecologists (ACOG), include the presence of new-onset hypertension, defined as a systolic blood pressure of at least 140 mmHg or a diastolic blood pressure of at least 90 mmHg after 20 weeks of gestation, with or without proteinuria. Proteinuria is diagnosed when there is evidence of at least 300 mg of protein in a 24 h urine collection, a protein/creatinine ratio of 0.3 or more, or a urine dipstick result of 2+ or greater [5]. In cases where proteinuria is absent, PE may still be diagnosed if other symptoms are present, including thrombocytopenia (a platelet count of less than 100 K/mm^3^), renal insufficiency (serum creatinine levels greater than 1.1 mg/dL), impaired liver function (elevation of liver transaminase levels to at least twice the normal range), or pulmonary edema [5,22]. PE is classified into two subtypes based on the timing of onset: early-onset PE, which occurs before 34 weeks of gestation, and late-onset PE, which occurs after 34 weeks. Women with late-onset PE generally experience more favorable maternal outcomes, with a lower likelihood of complications such as abruptio placentae and PE with severe features [23].

While ACOG emphasizes symptom-based diagnostic criteria, the Fetal Medicine Foundation (FMF) advocates for risk assessment at different stages of gestation. At weeks 11 + 0 to 14 + 6, screening focuses on identifying individuals at risk of preterm PE (<37 weeks) through maternal risk factors, the uterine artery pulsatility index (PI), mean arterial pressure (MAP), and PIGF levels [24,25]. Additional assessments at 19 + 0 to 25 + 6 and 30 + 0 to 35 + 6 weeks provide risk stratification for PE < 32 weeks and <36 weeks, categorizing patients into high-, intermediate-, and low-risk groups. High-risk individuals require vigilant monitoring of blood pressure and proteinuria between 24 and 31 weeks [24]. At 35 + 0 to 38 + 6 weeks, risk stratification informs monitoring strategies and decisions regarding the timing of delivery [24]. The FMF criteria emphasize a combined diagnostic approach to enhance predictive accuracy. Uterine artery PI alone has limited predictive value; however, when combined with MAP and biomarkers such as PIGF, its sensitivity improves significantly [26,27]. This integrative approach enables earlier detection and personalized management, potentially mitigating severe outcomes associated with PE.

**Figure 1:**
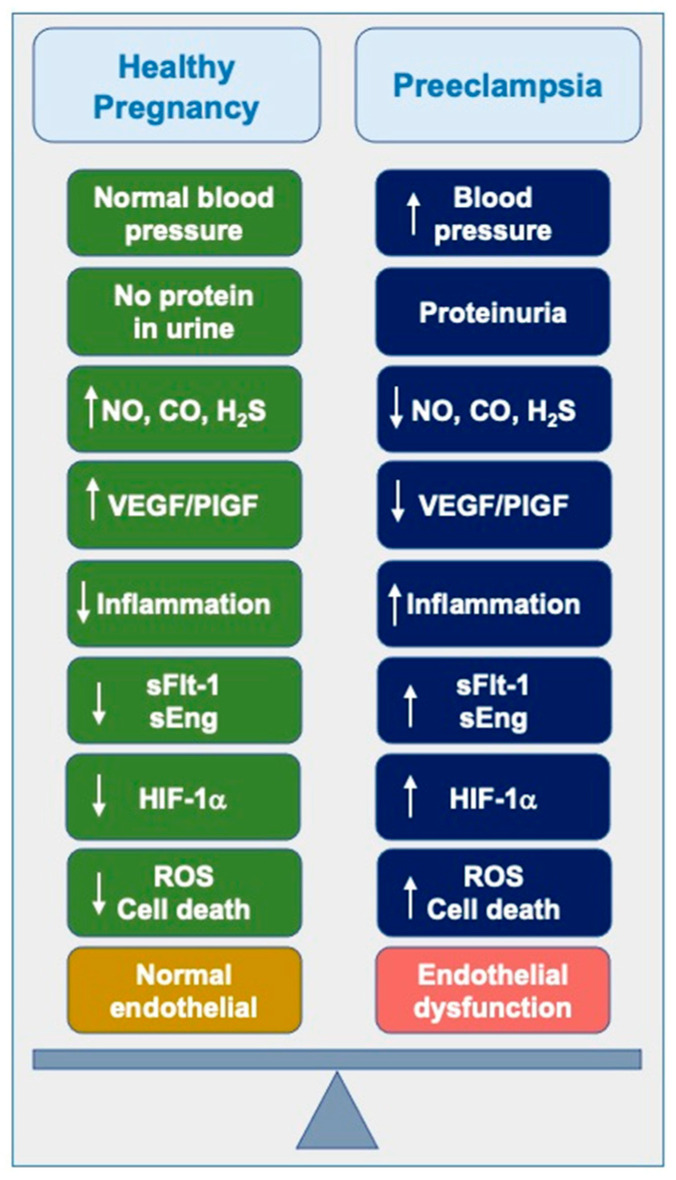
Pathogenesis of preeclampsia. High blood pressure and proteinuria are hallmarks of preeclampsia. Decreases in nitric oxide (NO) and hydrogen sulfide (H_2_S) lead to an increase in inflammation, the elevation of soluble Flt-1 (sFlt-1) and soluble Endoglin (sEng), and decreases in vascular endothelial growth factor (VEGF) and placenta growth factor (PlGF). Oxygen disruption leads to increases in hypoxia-inducible factor-1alpha (HIF-1a). These biochemical changes lead to the generation of reactive oxygen species (ROS), cell death, and endothelial dysfunction, which contribute to the pathogenesis of preeclampsia.

Emerging diagnostic methods have shown that biomarkers such as soluble fms-like tyrosine kinase-1 (sFlt-1) and soluble endoglin (sEng) can be effective in identifying PE. These antiangiogenic proteins are elevated in individuals with PE and suppress the activity of angiogenic factors like placental growth factor (PIGF) and vascular endothelial growth factor (VEGF) [28,29,30,31]. This imbalance contributes to hypertension, proteinuria, vasoconstriction, and endothelial damage [29,32,33]. The sFlt-1-to-PIGF ratio has gained attention as a predictive tool, particularly effective between 24 and 26 weeks of gestation [34]. Ratios below 38 indicate a low likelihood of developing PE, while ratios above 85 are strongly indicative of early-onset PE, and values between 38 and 85 suggest a moderate risk of PE that may require further monitoring [35,36,37]. These advancements in diagnostic measures provide valuable insights, particularly in the first and second trimesters, for predicting PE risk [34,38] (Figure 1). However, angiogenic markers alone cannot definitively diagnose or exclude PE. Instead, they are valuable for predicting disease progression, distinguishing PE from other hypertensive–proteinuric disorders, and identifying cases with severe features [39,40,41].

The primary strategies for managing PE include effective prenatal care, early diagnosis, and timely delivery of the fetus [42,43]. For late preterm PE, occurring between 34 and 37 weeks of gestation, early delivery has been shown to improve maternal outcomes significantly. A randomized clinical trial comparing early or planned delivery with expectant management revealed that early intervention through labor induction or cesarean section significantly reduced maternal mortality and complications related to hypertension [44]. Therefore, prenatal care plays a critical role in early detection, as routine blood pressure monitoring and urine testing can help identify the initial signs of PE, allowing for timely intervention. The current gold standard for the prevention and management of PE includes the use of aspirin and magnesium sulfate. The intrapartum management of PE focuses on blood pressure control and seizure prophylaxis to ensure maternal and fetal safety during labor. For acute severe hypertension, intravenous (IV) medications such as hydralazine, labetalol, or oral nifedipine are effective in lowering blood pressure promptly [5]. Magnesium sulfate remains the standard of care for seizure prophylaxis in women with PE, regardless of whether severe features are present [5].

A separate, yet equally important, subtype of PE is postpartum PE, which is defined as the new-onset of hypertension (>140/90 mmHg but <160/110 mmHg) within 48 h–6 weeks postpartum. Diagnostic criteria include proteinuria (protein/creatinine ratio of ≥0.3), thrombocytopenia (platelet count < 100,000), renal insufficiency (serum creatinine > 1.1 mg/dL or doubled creatinine levels in the absence of preexisting renal disease), impaired liver function (elevated liver transaminases to twice the normal level), or other severe features such as pulmonary edema, vision changes, or persistent headaches that are unresponsive to medication [45]. Management involves both acute and long-term measures. Short-acting antihypertensives, including IV labetalol, IV hydralazine, or oral nifedipine, are used to manage acute blood pressure elevation. Long-acting antihypertensives, such as oral labetalol or nifedipine, are employed for sustained control. Seizure prophylaxis is achieved with magnesium sulfate, and diuresis with oral furosemide may be required to manage fluid overload. Postpartum follow-up includes home blood pressure monitoring and routine postpartum visits [45]. Risk factors for postpartum PE include advanced maternal age, Black race, maternal obesity, a history of hypertensive disorders in prior pregnancies, and Cesarean delivery [45].

#### 2.2.1. The Role of Aspirin in Managing Preeclampsia

Aspirin is widely recommended as a preventive measure for women at high risk of developing PE. Multiple studies have demonstrated that daily aspirin regimens can significantly reduce the likelihood of PE onset [16]. Aspirin’s efficacy is attributed to its anti-inflammatory, antiangiogenic, and antiplatelet properties, which are beneficial in mitigating the risk factors associated with PE [16]. One of the key mechanisms by which aspirin helps prevent PE is by inhibiting soluble fms-like tyrosine kinase-1 (sFlt-1), a protein that impedes angiogenesis. By reducing sFlt-1 activity, aspirin supports proangiogenic factors and vascular health [16]. Moreover, aspirin irreversibly inhibits cyclooxygenase-1 (COX-1) and COX-2 enzymes, reducing prostacyclin levels and thereby modulating inflammatory responses [16].

**Figure 2:**
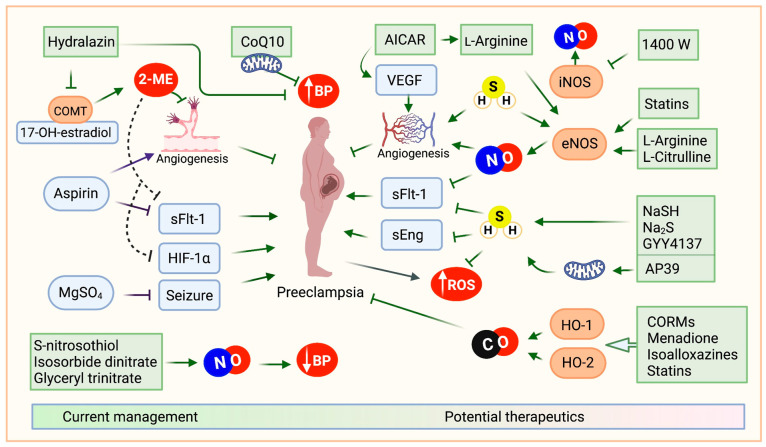
Current and future trends in preeclampsia management. Preeclampsia (PE) pathogenesis is linked to elevated levels of antiangiogenic factors such as sFlt-1 and sEng, which cause placental vasoconstriction, hypertension, and endothelial damage. Hypoxic stress in placental tissues triggers the release of hypoxia-inducible factors (HIFs), including HIF-1α, which mediate an acute adaptive response. Additionally, a reduction in COMT activity, which converts 17-hydroxyestradiol to the antiangiogenic 2-methoxyestradiol (2-ME), exacerbates the condition by sustaining elevated levels of HIF-1α and sFlt-1. Current therapeutic strategies include aspirin, which reduces sFlt-1 levels and promotes angiogenesis, hydralazine, which not only inhibits inositol triphosphate-induced calcium release but also suppresses placental COMT activity, and Coenzyme Q10 (CoQ10), recognized for its antioxidant properties and hypotensive effects. Emerging management approaches aim to leverage physiologic gasotransmitters such as nitric oxide (NO), carbon monoxide (CO), and hydrogen sulfide (H_2_S), which exhibit angiogenic effects and suppress sFlt-1 and sEng. To enhance NO levels, potential interventions include S-nitrosothiols, isosorbide dinitrate, glyceryl trinitrate, and precursors like L-arginine and L-citrulline, which target endothelial nitric oxide synthase (eNOS). For H_2_S, supplementation with compounds such as NaSH, Na_2_S, and GYY4137 has shown promise. CO levels can be augmented using CO-releasing molecules (CORMs) or compounds such as menadione, isoalloxazine, and statins, which stimulate heme oxygenase (HO-1/HO-2) enzymes to produce CO. These strategies reflect a convergence of pharmacologic and physiologic therapies aimed at reducing the impact of PE and improving maternal-fetal health outcomes.

The International Federation of Gynecology and Obstetrics (FIGO) advises a daily dosage of 150 mg of aspirin for high-risk patients starting between weeks 11 and 14 of gestation [16,46]. A meta-analysis conducted in 2018 revealed that women taking 150 mg of aspirin daily experienced a 62% reduction in the risk of developing PE. In comparison, those taking 81 mg of aspirin daily saw only a 30% reduction in risk [46]. ACOG provides additional guidelines, recommending low-dose aspirin (81 mg) for women with moderate PE risk factors, starting between 12 and 16 weeks of gestation [47]. For high-risk women, ACOG also supports daily low-dose aspirin administration within the same timeframe [47]. Initiating aspirin therapy after 12 weeks of pregnancy has been associated not only with a reduction in PE risk but also with decreased rates of preterm birth and fetal death [48], (Figure 2). Further research is necessary to evaluate the benefits and potential risks of initiating aspirin earlier in pregnancy (before 10 weeks). While recent studies suggest that aspirin may not be effective in reducing the incidence of superimposed PE, its ability to lower the risk of preterm birth justifies its continued prophylactic use in appropriate cases [49,50].

#### 2.2.2. Magnesium Sulfate in Managing Preeclampsia

ACOG strongly recommends the use of magnesium sulfate in the prenatal management of preeclampsia (PE) to prevent eclampsia, a serious complication involving seizures in pregnant women [9,21]. This therapeutic agent has become a cornerstone of treatment strategies due to its multifaceted mechanisms of action. Magnesium sulfate operates primarily as a vasodilator, reducing vascular resistance in the peripheral and cerebrovascular systems, thereby enhancing blood flow to the brain. This improved cerebral perfusion is critical in decreasing the risk of seizures. Moreover, magnesium sulfate stabilizes the blood–brain barrier, helping to prevent the formation of cerebral edema, a common and dangerous manifestation of eclampsia. Its central anticonvulsant effects are mediated through the inhibition of N-methyl-D-aspartate (NMDA) receptors, which are involved in excitatory neurotransmission and may contribute to the initiation of seizures in preeclampsia [48,51]. Clinical studies have demonstrated the efficacy of magnesium sulfate in significantly lowering the risk of eclampsia. For instance, preeclamptic women treated with magnesium sulfate experienced a 58% reduction in the likelihood of developing eclampsia compared to their untreated counterparts [51] (Figure 2). Furthermore, magnesium sulfate has been assessed in multiple meta-analyses for its neuroprotective effects in preterm infants. These studies found that its administration notably reduces the risk of cerebral palsy and other neurological impairments in neonates born prematurely, underscoring its dual benefits for both maternal and fetal health [52,53,54]. When compared to other anticonvulsant medications, such as diazepam and phenytoin, magnesium sulfate demonstrates superior efficacy in preventing recurrent seizures and reducing maternal mortality rates. This comparative advantage has solidified its position as the first-line treatment for seizure prophylaxis in preeclamptic women [55].

The current guidelines for magnesium sulfate administration in seizure prophylaxis recommend an intravenous loading dose of 4–6 g, delivered over 20–30 min, followed by a continuous maintenance infusion of 1–2 g per hour. This dosing regimen provides effective seizure prevention while minimizing the potential for adverse effects [5]. Despite its proven efficacy, magnesium sulfate is not without risks, particularly for neonates exposed to prolonged treatment. Extended maternal administration (beyond 5–7 days) has been associated with neonatal complications such as osteopenia and bone fractures. These adverse effects are thought to result from magnesium’s interference with calcium metabolism, emphasizing the importance of adhering to recommended treatment durations [56]. Ongoing research aims to refine magnesium sulfate therapy, exploring optimal dosing regimens to maximize benefits while minimizing risks. Studies examining whether magnesium sulfate could be used alongside other therapies, such as aspirin or antihypertensives, to further enhance outcomes for high-risk pregnancies are lacking to the best of our search.

### 2.3. Inhibition of COMT and Suppression of Hypoxia-Inducible Factors (HIFs)

Hypoxia-inducible factors (HIFs) are transcription factors that become active under conditions of low oxygen tension, playing a critical role in cellular adaptation to hypoxia [57]. The cellular hypoxic response is mediated by the HIFα subunits, which dimerize with the beta subunit, known as the aryl hydrocarbon receptor nuclear translocator (ARNT). This complex binds to hypoxia-responsive elements (HREs) in target genes to activate transcription [58]. Three HIFα isoforms have been identified: HIF-1α, HIF-2α, and HIF-3α. While HIF-1α mediates the acute response to hypoxia, HIF-2α predominantly governs chronic hypoxic adaptations [59]. The function of HIF-3α remains less understood, although evidence suggests it may negatively regulate hypoxia-responsive genes [59,60]. In preeclamptic women, HIF-1α expression is markedly elevated in placental tissues, reflecting the chronic hypoxic environment caused by insufficient cytotrophoblast invasion and defective uterine spiral artery remodeling, leading to placental ischemia [9,14,61,62].

Catechol-O-Methyltransferase (COMT), an enzyme responsible for degrading catecholamines and catechol estrogens, has been implicated in preeclampsia pathogenesis. COMT converts 17-hydroxyestradiol into 2-methoxyestradiol (2-ME), a bioactive molecule with potent antiangiogenic properties that suppresses both HIF-1α and sFlt-1 expression [14,63,64]. COMT-deficient mice exhibit a preeclampsia-like phenotype characterized by placental hypoxia, increased nuclear HIF-1α levels in the placenta, and elevated circulating sFlt-1 [64]. Functional polymorphisms in human COMT can result in reduced enzymatic activity at physiological temperatures, contributing to fetal growth restriction and other complications of preeclampsia [63,64]. Treatment with 2-ME in COMT-deficient mice has been shown to reverse placental hypoxia, normalize HIF-1α activity, and lower circulating sFlt-1 [64].

Hydralazine, a vasodilator commonly used to treat severe hypertension during pregnancy, acts by inhibiting inositol trisphosphate-induced calcium release in arterial smooth muscle cells [65]. Although effective, hydralazine has been shown to inhibit placental COMT activity, potentially exacerbating preeclampsia by reducing 2-ME levels. Furthermore, hydralazine has been associated with adverse outcomes such as placental abruption in some studies [14,66], a complication that often results in maternal and fetal morbidity and mortality. These findings highlight the need for cautious evaluation of hydralazine use in PE management [14].

Alternatives to hydralazine include nifedipine, a calcium channel blocker commonly used for hypertension and angina; ketanserin, a serotonergic receptor antagonist that inhibits serotonin-mediated vasoconstriction and platelet activation; and labetalol, an α1- and non-selective β-blocker [66,67] (Figure 2). Among these, labetalol’s parenteral formulation is particularly useful for rapid blood pressure reduction in emergencies.

### 2.4. CoQ10 Supplementation

Coenzyme Q10 (CoQ10) is a fat-soluble vitamin-like molecule synthesized endogenously from phenylalanine and meyalonic acid, and it is also obtained from the diet [15]. It plays a crucial role in mitochondrial respiration, particularly within the electron transport chain, as it facilitates electron transfer between complexes I, II, and III [15]. CoQ10 also serves as a potent antioxidant, protecting cellular components from oxidative damage caused by reactive oxygen species (ROS). Deficiencies or genetic mutations affecting CoQ10 can impair ATP production, increase oxidative stress, and disrupt cellular energy balance. In PE, a significant decrease in CoQ10 levels has been observed when compared to normal pregnancies [68]. This reduction in CoQ10 contributes to mitochondrial dysfunction and increased oxidative stress, both of which play key roles in the pathophysiology of PE. Research suggests that CoQ10 supplementation can mitigate these effects by improving mitochondrial efficiency and reducing oxidative damage. Clinical trials have demonstrated the potential benefits of CoQ10 supplementation for reducing the incidence of PE. In a randomized, double-blind study, the prophylactic administration of 100 mg of CoQ10 daily from 20 weeks of gestation significantly decreased the risk of developing PE in women identified as being at high risk. The risk was reduced from 25.6% in the placebo group to 14.4% in the CoQ10-treated group [68]. This suggests a notable protective effect against the development of PE. Beyond its implications for PE, CoQ10 supplementation has been shown to lower blood pressure, reduce the risk of diabetes, and improve outcomes in patients with cardiovascular diseases [69,70,71] (Figure 2). These benefits are particularly relevant in the context of PE, given the overlap in pathophysiological mechanisms between PE and cardiovascular disorders.

## 3. Experimental Treatments of Interest

Currently, there is no definitive cure for PE. However, several potential approaches have emerged based on robust data-driven research, which could lead to improved management and outcomes.

### 3.1. Restoration of Angiogenic Balance

In PE, an imbalance between antiangiogenic and proangiogenic factors is a key pathological feature. Women with PE exhibit elevated levels of antiangiogenic factors, including sFlt-1 and sEng, which disrupt the vascular equilibrium necessary for healthy placental development [72]. These antiangiogenic factors reduce the levels of vascular endothelial growth factor (VEGF) and placental growth factor (PIGF), both of which are essential for endothelial cell survival and vascular permeability during pregnancy [73]. Efforts to restore angiogenic balance focus on modulating these factors to alleviate maternal symptoms such as hypertension and endothelial dysfunction.

### 3.2. Nitric Oxide-Donors

Nitric oxide (NO) is an important signaling molecule that has a pivotal role in vascular regulation, lowering blood pressure, dilating blood vessels, and enhancing placental blood flow [74]. Alterations in NO production in the feto–placental unit have been implicated in the vasoconstriction and other hallmark symptoms of PE [1].

NO is synthesized from L-arginine by the enzyme nitric oxide synthase (NOS), of which there are three different isoforms: neuronal (nNOS/NOS1), endothelial (eNOS/NOS3), and inducible (iNOS/NOS2), reviewed in [75]. The expression levels of these enzymes vary in different tissues; nNOS and eNOS produce low concentrations of NO for short periods, whereas iNOS produces relatively higher levels for longer periods. eNOS is crucial during pregnancy as it promotes cytotrophoblast invasion, implantation, and placental perfusion [76]. However, in PE, abnormalities in the eNOS-NO system are reported. Some studies indicate decreased eNOS activity, which leads to vasoconstriction of the placental bed, renal vasculature, and other organs [8,77]. Conversely, other findings suggest increased eNOS expression in PE, potentially functioning as a compensatory mechanism to counteract maternal vascular dysfunction [76].

The significance of iNOS expression in PE is also controversial. Some studies associate increased iNOS levels with hypertension and oxidative stress, indicating potential benefits for iNOS inhibition [78,79] (Figure 2). However, other studies find no significant difference in iNOS expression between PE and normal pregnancies [78].

NO-donating compounds show promise for preventing and treating PE. Glyceryl trinitrate and isosorbide dinitrate reduce uterine artery resistance and maternal blood pressure but can cause tolerance with continuous use, necessitating nitrate-free intervals [80] (Figure 2). Pentaerythryl tetranitrate reduces fetal growth restriction and preterm birth, while S-nitrosothiols lower blood pressure, uterine artery resistance, and platelet activation in severe PE cases [80]. Interestingly, mice lacking the S-nitrosoglutathione reductase (GSNOR) gene, a denitrosylase, which contributes to increase nitrosylated proteins in circulation, have shown PE-like phenotypes, including hypertension, proteinuria, fetal growth restriction, and abnormal cardiovascular and renal adaptions [81]. Supplementing with L-arginine and L-citrulline has been shown to increase NO levels and improve feto–placental blood flow [76,82] (Figure 2). The combined administration of these amino acids further enhances NO production, offering a potential therapeutic strategy [83].

Inhibiting e-NOS can have harmful effects in PE. Elevated levels of asymmetric dimethylarginine (ADMA), an endogenous inhibitor of eNOS, are observed in PE and correlate with impaired endothelial function and oxidative stress. ADMA, which is produced via the methylation of arginine residues within specific proteins by a family of enzymes called protein arginine methyltransferases, inhibits eNOS-NO synthesis, exacerbating vascular dysfunction and oxidative stress in PE [1,84]. Addressing this disruption in the NO pathway, through NO donors or by enhancing NO signaling, represents an attractive therapeutic approach [1,8].

AMPK (AMP-activated protein kinase) activation also offers promise. AICAR (5-aminoimidazole-4-carboxamide-3-ribonucleoside), an AMPK agonist, increases VEGF expression, reduces oxidative stress, and restores the ADMA-NO pathway [85], thus preventing hypertension in PE patients [86]. In addition to increasing VEGF concentrations, AICAR can also restore the ADMA-NO pathway [86]. While AICAR has many benefits, at high concentrations, it has been found to be toxic to mammalian cells, thus requiring further investigation [86] (Figure 2).

Although advancements have been made, challenges persist in clarifying the roles of eNOS and iNOS in PE and optimizing therapeutic strategies. Future research should focus on refining NO-donor therapies, personalizing treatment approaches, and exploring combination therapies involving L-arginine, L-citrulline, and AMPK agonists to improve outcomes for patients with PE.

### 3.3. Modulation of Carbon Monoxide Levels

Carbon monoxide (CO) is a colorless, odorless gas that is most infamous as a “silent killer” because it is hard to detect, and its intoxication can be fatal. Although highly toxic, CO has many physiological roles when produced endogenously [87]. The primary source of endogenous CO is the breakdown of heme by the enzyme heme oxygenase (HO) [88], of which there are three isozymes: HO-1 is inducible and is expressed in response to cellular stress [89,90,91]; HO-2 is constitutively expressed, and its function is associated with neurotransmission and the regulation of vascular tone [92,93]; and HO-3, which is also constitutive but functionally enigmatic due to its lack of heme-degrading activity [94,95]. CO, like NO, activates guanylyl cyclase to produce cGMP (cyclic GMP), albeit with much lower efficacy (1/80th that of NO) [94]. In addition, low CO concentrations activate K_ATP_ channels and affect the MAPK signaling pathways [96].

As an endogenously produced gasotransmitter, CO has a role in inflammation, apoptosis, angiogenesis, and vascular tone [97,98,99]. Women who smoke—a behavior associated with increased CO levels—exhibit a 33% reduced risk of developing PE [100,101], which correlates with reduced circulating sFlt-1 levels [102,103]. Lower end-tidal breath CO levels in preeclamptic women compared to healthy pregnancies suggest a potential contribution of reduced CO to PE pathogenesis [104]. Furthermore, studies show that HO-1 [105] and HO-2 [106,107] protein levels are decreased in the preeclamptic placenta. Experimental overexpression of HO-1 in endothelial cells reduces sFlt-1 and sEng levels, whereas silencing HO-1 via siRNA enhances their release [108]. However, one study reported no significant decrease in HO-1 expression in preeclamptic placentas and found no evidence of HO-1 regulating placental sFlt-1 or sEng secretion [109].

In a rat model of placental ischemia, HO-1 induction restored angiogenic balance, reduced blood pressure, and inhibited hypoxia-induced sFlt-1 production in vitro [110]. In pregnant CD-1 mice, chronic CO exposure increased placental vessel branching, arterial size, and perfusion without adverse maternal or fetal effects [111,112,113]. These findings suggest therapeutic potential for modulating CO levels in PE.

Efforts to pharmacologically elevate CO have explored multiple strategies. Synthetic vitamin K3 (Menadione, MD) significantly enhances CO production in vitro using rat spleen and brain microsomes by increasing HO-2 activity [114] and NADPH–cytochrome P-450 oxidoreductase (CPR) [115]. Of note, vitamins K_1_ and K_2_, were not able to activate HO-2 since they are more lipophilic than MD [114]. MD also boosts CO levels in isolated GD15 placentas [116]. Furthermore, MD also boosts CO levels in the liver, spleen, and placenta in pregnant mice, with potential applications for PE management.

Pyrroloquinoline quinone (PQQ), an aromatic tricyclic ortho-quinone found in various foods, exhibits significant anti-inflammatory properties [117]. Similarly, isoalloxazine, a key structural component of flavin molecules such as riboflavin (vitamin B2), flavin mononucleotide (FMN), and flavin adenine dinucleotide (FAD), also plays a pivotal role in biochemical processes and serves as a potent stimulator of CO production [98]. Studies using rat spleen and brain tissues, as well as human placenta microsomes, demonstrated that both PQQ and riboflavin significantly enhance CO production. This effect extends to systems employing purified recombinant human cytochrome P450 reductase (rh-CPR), indicating their ability to facilitate CO generation through electron transfer mechanisms [98]. These findings highlight the functional parallels between PQQ and flavins in modulating CO levels, which may underlie their anti-inflammatory and antioxidant effects.

Direct CO inhalation is an effective delivery method but impractical. Therefore, CO-releasing molecules (CORMs) have been developed, delivering CO in a dose-dependent manner; they are categorized into metal carbonyls and nonmetallic CORMs; for details, please see [118]. The administration of CORM-3, a soluble CO donor to rats from GD14 to GD19, significantly reduced the hypertensive response to placental ischemia on GD19 by increasing the glomerular filtration rate, though it does not directly affect sFlt-1 levels in the placenta or maternal circulation [110,119]. Similarly, CORM-2 reduces sFlt-1 release in preeclamptic villous explants by inhibiting VEGFR-2 phosphorylation [108]. CORM-A1, a water-soluble CO donor with vasodilatory effects, does not alter blood pressure or heart rate in normotensive pregnant mice [89,120].

These findings highlight CO’s therapeutic potential in restoring angiogenic balance, reducing oxidative stress, and improving placental perfusion in PE. However, the variability in results regarding sFlt-1 modulation underscores the need for further studies to refine CO-based interventions.

### 3.4. H_2_S-Donors

Hydrogen sulfide (H_2_S) is a critical gasotransmitter involved in various physiological processes, including vasodilation, angiogenesis [121], and the resolution of inflammation [122]. Its endogenous production stems from direct cysteine desulfhydration by the enzymes cystathionine-γ-lyase (CSE, EC 4.4.1.1), cystathionine-β-synthase (CBS, EC 4.2.1.22), and indirect desulfhydration by 3-mercapto-sulfurtransferase (3-MST, EC 2.8.1.2) in tandem with cysteine aminotransferase (CAT, EC 2.6.1.3) [122]. Though CBS, CSE, and MST are constitutive enzymes with different expression levels in various tissues and organs [34], recent studies suggest that CSE may be inducible [121].

CBS and CSE are expressed in the placentas of healthy pregnant women [123]; however, in PE, their expression is significantly reduced [11,121], leading to lower circulating H_2_S levels [9]. This reduction contributes to oxidative stress and impaired vascular function. Although early-onset PE villous tissue shows unaltered CSE expression, CBS levels are markedly diminished [124].

The therapeutic administration of H_2_S donors has shown promise in addressing oxidative stress. Sulfide salts, such as sodium sulfide (Na_2_S) and sodium hydrosulfide (NaSH), provide rapid H_2_S release, which mitigates damage from reactive oxygen species (ROS) [125,126]. These compounds have demonstrated efficacy in neurodegenerative models and oxidative stress management [127], including hepatic ischemia–reperfusion injury [128]. Additionally, treatment with sulfide salts reduces sFlt-1 and proteinuria [9,124], (Figure 2). However, their therapeutic potential is limited by rapid oxidation and instantaneous H_2_S release [129].

Slow-releasing H_2_S donors like GYY4137 [130] overcome these limitations by providing sustained release. GYY4137 enhanced fetal growth that was compromised by CSE inhibition and inhibited the rise in circulating sFlt-1 and sEng levels [11] that are crucial factors in PE pathogenesis. It also increased eNOS activity, thereby increasing NO levels [131]. In a study evaluating the utility of GYY4137 in managing necrotizing enterocolitis, animals treated with GYY4137 gained weight and had decreased sickness scores, which was accompanied by vasodilation and increased blood flow [131]. Moreover, it promotes VEGF production, which is typically suppressed in PE [131]. Overall, as a H_2_S donor and eNOS modulator, GYY4137 has many potential benefits that may ultimately contribute to PE management (Figure 2).

AP39, a mitochondria-targeted H_2_S donor, addresses mitochondrial dysfunction prevalent in PE. It improves mitochondrial bioenergetics and reverses mitochondrial oxidative stress [10]. In preeclamptic patients, the activity of the mitochondrial electron transport chain at complex IV is reduced [132]. This leads to lower oxidative phosphorylation and mitochondrial respiration in the preeclamptic placenta. When the mitochondria are unable to produce an antioxidant response sufficiently, ROS builds up, which can be quite toxic. Treatment with AP39 reduced ROS levels and diminished sFlt-1 production [10]. In addition, AP39 protects mitochondrial DNA and protects endothelial cells against oxidative stress [133] (Figure 2). The effects of AP39 are bell-shaped. At low concentrations (30–100 nM), mitochondrial protection is observed, whereas at relatively higher concentrations (300 nM), it is less effective or not effective at all [133,134].

In conclusion, the diverse mechanisms of action exhibited by H_2_S donors such as sulfide salts, GYY4137, and AP39 position them as promising candidates for treating PE and other oxidative stress-related conditions. These compounds not only mitigate ROS-induced damage but also restore vascular and mitochondrial functions, offering a multifaceted approach to therapy.

### 3.5. Statins

Statins are a class of widely used cholesterol-lowering drugs that work by competitively and reversibly inhibiting HMG-CoA reductase, the rate-limiting enzyme in hepatic cholesterol biosynthesis. This mechanism reduces serum levels of LDL cholesterol (LDL-C), triglycerides, and total cholesterol [135,136,137]. Statins are categorized into two types: lipophilic (e.g., cerivastatin, simvastatin, lovastatin, fluvastatin, and atorvastatin) and hydrophilic (e.g., pravastatin and rosuvastatin) [138]. Beyond their cholesterol-lowering properties, statins exhibit pleiotropic effects, contributing to their role in vascular health [139,140]. For example, they upregulate eNOS, thus resulting in higher production of endothelial NO levels, which plays a critical role in vasodilation, platelet aggregation, vascular smooth muscle proliferation, and reducing endothelial–leukocyte interactions [139,141]. Statins achieve eNOS upregulation through mechanisms such as the modulation of Rho/ROCK signaling and the activation of the PI3K/Akt pathway, which phosphorylates eNOS [142]. Interestingly, statins also exhibit dose-dependent effects on angiogenesis, promoting it at low doses and inhibiting it at higher doses [143].

In pregnancy-related conditions such as PE, statins have shown potential benefits in preclinical and clinical settings [144]. Pravastatin, in particular, has been associated with improved pregnancy outcomes in animal models of PE. Its effects include reducing inflammation, enhancing placental blood flow, and correcting angiogenic and redox imbalances [145,146]. This leads to a reduction in blood pressure and the stabilization of proteinuria, as observed in pravastatin-treated preeclamptic patients. Notably, pravastatin has been linked to the upregulation of VEGF and placental growth factor (PlGF), which contribute to placental vascularization and function [147]. Simvastatin has also demonstrated the ability to inhibit sFlt-1 production, further supporting the potential role of statins in PE management. Clinical case reports suggest that pravastatin treatment in preeclamptic patients resulted in improved placental blood flow and the stabilization of hypertension and proteinuria, with live births achieved [8,73,148] (Figure 2).

Despite their promise, statins are not without risks, including myopathy, memory loss, and a potential increase in new-onset diabetes [137]. However, these concerns are primarily associated with long-term use and are less significant in the short-term treatment of conditions such as PE. Thus, statins remain a compelling area of research for improving maternal and fetal outcomes in preeclampsia.

Current therapeutic strategies include aspirin, which reduces sFlt-1 levels and promotes angiogenesis; hydralazine, which not only inhibits inositol triphosphate-induced calcium release but also suppresses placental COMT activity; and Coenzyme Q10 (CoQ10), recognized for its antioxidant properties and hypotensive effects.

Emerging management approaches aim to leverage physiologic gasotransmitters such as nitric oxide (NO), carbon monoxide (CO), and hydrogen sulfide (H_2_S), which exhibit angiogenic effects and suppress sFlt-1 and sEng. To enhance NO levels, potential interventions include S-nitrosothiols, isosorbide dinitrate, glyceryl trinitrate, and precursors like L-arginine and L-citrulline, which target endothelial nitric oxide synthase (eNOS). For H_2_S, supplementation with compounds such as NaSH, Na_2_S, and GYY4137 has shown promise. CO levels can be augmented using CO-releasing molecules (CORMs) or compounds such as menadione, isoalloxazine, and statins, which stimulate heme oxygenase (HO-1/HO-2) enzymes to produce CO.

These strategies reflect a convergence of pharmacologic and physiologic therapies aimed at reducing the impact of PE and improving maternal–fetal health outcomes.

## 4. Preeclampsia and Racial Disparities

Racial disparity encompasses imbalances in treatment, opportunities, and outcomes between racial groups in areas like economic status, housing options, healthcare access, and societal treatment [149]. In maternal health, racial disparities are glaring, with maternal race being a significant risk factor for severe maternal morbidity and mortality [150]. Black women, in particular, face disproportionately higher risks for maternal mortality and PE, a trend that has persisted over the past 50 years [151,152,153,154,155]. This disparity correlates with the higher prevalence of hypertension and cardiovascular disease among Black women during pregnancy [151]. Black women are at higher risk for stroke, renal failure, and overall death during delivery than other races [154].

### 4.1. Maternal Mortality and PE Disparities

In-hospital maternal mortality rates for Black women during antepartum, intrapartum, and postpartum periods are strikingly higher than those for other racial groups, with rates of 47, 17, and 379 per 100,000 hospitalizations, respectively. In comparison, white women have respective rates of 30, 5, and 160 per 100,000, while Hispanic women show rates of 28, 6, and 181 per 100,000 hospitalizations [156].

**Figure 3:**
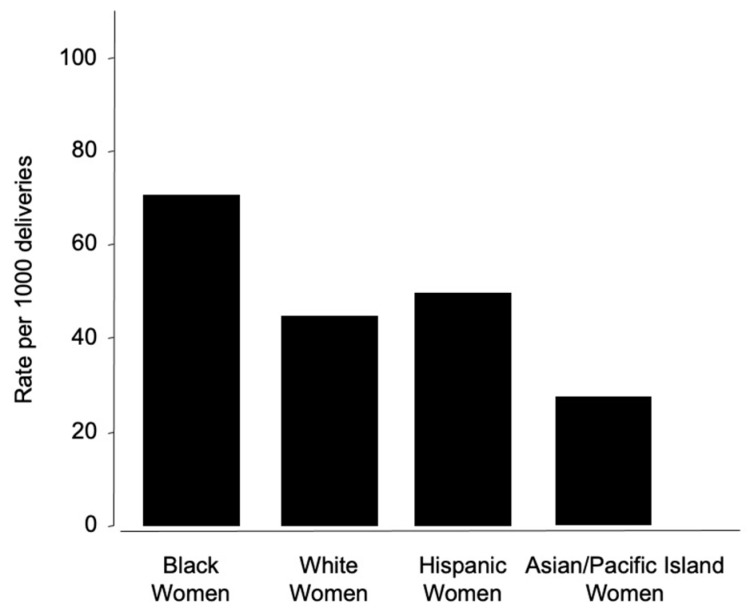
Racial disparities of preeclampsia. Preeclampsia rates within the United States demonstrate significant racial disparities when measured per 1000 deliveries. Black women experience preeclampsia at the highest rate, approximately 69.8 cases per 1000 deliveries. In contrast, white women have a rate of 43.3 per 1000 deliveries. Hispanic women experience preeclampsia at a slightly higher rate of 46.8 per 1000 deliveries, while Asian or Pacific Islander women have the lowest recorded rate at 28.8 per 1000 deliveries.

The incidence of preeclampsia further underscores the disparity. Within the US, Black women experience PE at a rate of 69.8 per 1000 deliveries, compared to 43.3 per 1000 for white women, 46.8 per 1000 for Hispanic women, and 28.8 per 1000 for Asian or Pacific Islander women [157] (Figure 3). In a retrospective cohort study that analyzed women with a diagnosis of PE, Hispanics were more likely to have severe PE compared to other races (40.4%), and whites were more likely to have mild PE (52.9%) [158]. Interestingly, a study examining the differential risks of hypertensive disorders during pregnancy amongst Hispanic women found that they had significantly lower rates of gestational hypertension than Caucasian women, even though the Hispanic women had a lower maternal socioeconomic status [159]. Native American women also face a 17% increased risk of PE compared to white women [160].

These data emphasize the critical need for addressing racial disparities in maternal healthcare to improve outcomes for high-risk groups

### 4.2. Genetic and Environmental Factors

Black individuals are more likely to carry variations in the *APOL1* gene, which is linked to an increased risk of kidney disease and pregnancy complications, including PE [161,162]. APOL1-related complications can affect maternal health, especially when expressed in the infant, further exacerbating disparities in outcomes.

Globally, the burden of PE is even greater in developing countries, where according to the World Health Organization (WHO), the incidence is seven times higher than in developed nations [163]. In third world countries where healthcare agencies and equipment are sparse, the highest rates of PE are observed. For example, worldwide, the incidence of PE is 3–5%, whereas in India, it ranges from 7.4 to 11.3%, contributing to 36% of all preterm births [164]. In Latin America and the Caribbean, hypertensive pregnancy disorders cause 26% of maternal deaths. Limited access to healthcare infrastructure, lack of awareness, and delayed treatment are critical factors driving these disparities [165].

### 4.3. Systemic Racism in Medicine

Racism at systemic and individual levels significantly impacts the quality of care for people of color. Black communities often lack access to high-quality medical facilities due to economic, political, and social barriers. Socioeconomic status, including income, education, and social class, directly correlates with health outcomes [166]. These disparities are exacerbated in poorer nations, where healthcare systems are underdeveloped, resulting in delayed or inadequate treatment for conditions like PE.

### 4.4. Addressing Racial Disparities

Addressing these disparities requires a multipronged approach, including (i) increasing access to quality maternal healthcare for underserved populations, (ii) promoting awareness and education about PE and its risk factors, (iii) strengthening healthcare systems in developing countries, and (iv) tackling systemic racism and socioeconomic barriers to healthcare.

By addressing the root causes of these disparities, significant strides can be made in reducing maternal mortality and improving pregnancy outcomes for all racial and ethnic groups.

## 5. Concluding Remarks and Perspectives

While significant progress has been made in understanding the pathophysiology of PE, its clinical management remains a substantial challenge. Current preventive strategies, both pharmacological and non-pharmacological, show only limited efficacy. To develop more effective treatments, there is a pressing need for improved animal models that can replicate the complex mechanisms of PE. Advanced genetic tools, such as tissue-specific knockout and knockdown mouse models, could provide deeper insights and more precise testing platforms [167,168]. Furthermore, basic research remains essential to unravel the intricacies of PE pathophysiology, which could unlock novel therapeutic targets [169].

Finally, socioeconomic status, a lack of education, and medical racism have significant bearings on the increased risk of PE in minority populations, limiting access to healthcare [170]. Alongside efforts to develop future management strategies for PE, healthcare providers must address the immediate issue of racial disparities within the healthcare system. Structural and systemic racism is deeply ingrained in society. However, current and future healthcare providers can practice measures to limit their own biases and build better relationships with their patients [171]. These measures include practicing effective empathetic communication with patients, becoming culturally competent, and acknowledging the barriers created by racism. By integrating these practices, healthcare providers can help mitigate disparities and improve maternal health outcomes across diverse populations

## Data Availability

Not applicable.

## Abbreviations

2-ME	2-methoxyestradiol
ACOG	American Congress of Obstetricians and Gynecologists
ADMA	asymmetric dimethylarginine
AICAR	5-aminoimidazole-4-carboxamide-3-ribonucleoside
AMPK	AMP-activated protein kinase
APOL1	Apolipoprotein L1
CBS	Cystathionine-β-synthase
COMT	Catechol-O-Methyltransferase
CoQ10	Coenzyme Q10
CSE	Cystathionine γ-lyase
eNOS	endothelial NOS/Type 3
FIGO	Federation of Gynecology and Obstetrics
HIF-1α	Hypoxia-inducible factor 1α
HIF-2α	Hypoxia-inducible factor 2α
HIF-3α	Hypoxia-inducible factor 3α
HMG-CoA	β-Hydroxy β-methylglutaryl-CoA
HRE	Hypoxia responsive elements
iNOS	inducible NOS/Type 2,
IUGR	Intrauterine growth
Na_2_S	Sodium sulfide
NaSH	Sodium hydrosulfide
NEC	Necrotizing enterocolitis
nNOS	neuronal NOS/Type 1,
NO	Nitric oxide
PIGF	Placental growth factor
ROS	Reactive oxygen species
sEng	Soluble endoglobin
sFlt-1	Soluble fms-like tyrosine kinase 1
VEGF	Vascular endothelial growth factor
